# Interobserver Reliability of the RENAL Nephrometry Scoring System: Experience From a Developing Nation

**DOI:** 10.7759/cureus.11451

**Published:** 2020-11-11

**Authors:** Kumail Khandwala, Dawar B Khan, Zainab Hussain, Nida Sajjad, Muhammad Ismail Alvi

**Affiliations:** 1 Radiology, Aga Khan University Hospital, Karachi, PAK

**Keywords:** clear-cell renal carcinoma, r.e.n.a.l nephrometry score, computed tomography

## Abstract

Introduction

The RENAL (radius, exophytic/endophytic properties, nearness of tumor to the collecting system or sinus in millimeters, anterior/posterior, location relative to polar lines) nephrometry score (RENAL-NS) has been described as a structured and quantiﬁable method to describe a renal tumor’s relevant anatomic features as they relate to the complexity of the lesion. We aim to evaluate a tumor’s RENAL-NS and to assess the reproducibility of the score among different observers.

Methods

This retrospective study included 49 patients diagnosed with renal cell carcinoma (RCC) who had complete computed tomography (CT) data, RENAL-NS, and histopathology results. All patients underwent renal surgery/intervention at our center between January 2008 and December 2018. The radius of the lesion, exophytic/endophytic properties, nearness to the collecting system, anterior or posterior description, and location relative to the polar lines was used to calculate the score. Tumor complexity was graded as low, intermediate, or high. Two body imaging radiologists evaluated the data independently.

Results

Interobserver agreement for each of the RENAL-NS parameters, respectively, and overall complexity was calculated. The total agreement was 82%, 51%, 84%, 69%, 73%, and 90%, corresponding to Kappa values of 0.72, 0.33, 0.44, 0.49, 0.58, and 0.83, respectively. The radius, nearness to the collecting system, and total complexity showed the best agreement. Exophytic properties of the lesion showed the least agreement. For cases that were discordant in terms of the final score, no major implications in surgical planning were observed.

Conclusion

The results of this study show that the RENAL-NS is a useful tool to assess the anatomical features of renal tumors and it is easily reproducible, even for less experienced radiologists in a developing nation.

## Introduction

Renal cell carcinoma (RCC) represents 2%-3% of all cancers, with the highest incidences occurring in Western countries [1]. A rising incidence in smaller-sized tumors has been noted in the last decade due to improvement in cross-sectional imaging. A local study in Pakistan showed a frequency of RCC ranging between 1.5% and 1.8% of all malignancies [2].

Cross-sectional imaging techniques allow the accurate evaluation of the tumor characteristics, its relationship with the adjacent structures, and the percentage of renal parenchymal involvement. This radiological information is of critical significance to urologists for planning surgical or ablative management [3]. Previously, multiple attempts to formulate a noninvasive method to predict the characteristics of renal masses, including their association with age, gender, symptoms, and smoking history have been carried out; however, their success has been limited.

Recently, the RENAL (radius, exophytic/endophytic properties, nearness of tumor to the collecting system or sinus in millimeters, anterior/posterior, location relative to polar lines) nephrometry score (RENAL-NS) has been introduced as a tool to describe the anatomic features of renal masses seen on cross-sectional imaging in attempts to predict outcomes and develop standardized management plans [3]. The RENAL-NS is a structured and quantiﬁable method to describe the tumor’s relevant anatomic features as they relate to the complexity of a tumor. 

To the best of our knowledge, authentic data to validate the RENAL-NS system is lacking in a developing country like Pakistan. From our literature search, only a single study has been conducted recently to determine the interobserver variability of RENAL-NS from Lahore, Pakistan [4]. Therefore, in the present study, we aim to evaluate a tumor’s RENAL-NS and to assess the reproducibility of the score among two different observers.

This article was presented as an electronic poster at the European Congress of Radiology virtual meeting held online from July 15 to July 19, 2020.

This article was also previously presented as an abstract: Kumail Khandwala, Zainab Hussain, Dawar Burhan Khan. Interobserver Reliability of the R.E.N.A.L. Nephrometry Scoring System: Preliminary Experience from a Developing Nation. European Congress of Radiology in Vienna, Austria; July 15-19, 2020.

## Materials and methods

Our retrospective, single-center study was approved by the institutional Ethical Review Committee of Aga Khan University, Karachi, Pakistan, and, therefore, the need for informed consent was waived. This study enrolled 49 patients with RCC who had complete computed tomography (CT) data and underwent renal intervention at our center between January 2008 and December 2018 with available histopathology results. Renal surgery consisted of nephron-sparing surgery or partial nephrectomy and radical nephrectomy/cytoreductive nephrectomy. Other interventional procedures like angiographic embolization of renal arteries were also recorded. Only unilateral, unifocal, and pathologically confirmed RCCs were included. Unilateral multifocal tumors, bilateral multifocal tumors, and cystic renal tumors were excluded.

An RCC stage was assigned by surgical pathology based on the American Joint Committee on Cancer (AJCC) 2010 tumor-node-metastasis (TNM) classification. Fuhrman grade I and II were classified as low Fuhrman grade (LFG), and Fuhrman grade III and IV were classified as high Fuhrman grade (HFG).

For the CT examinations, we used a 64 or 640-slice multi-detector CT (MDCT) (Toshiba Aquilion Series; Canon Medical Systems Corporation, Tochigi, Japan). All studies involved pre-contrast and dynamic post-contrast acquisition (arterial, venous, and equilibrium phases), a 40-second delay for the arterial phase, then an 80-second delay for the venous phase, and a two to three minutes’ delay for the delayed/excretory phase. Nonionic contrast media was used according to patient weight (1-2 mL/kg). Images were acquired with 3 mm slice thickness and reconstruction 5 mm in the sagittal and coronal planes.

**Figure 1:**
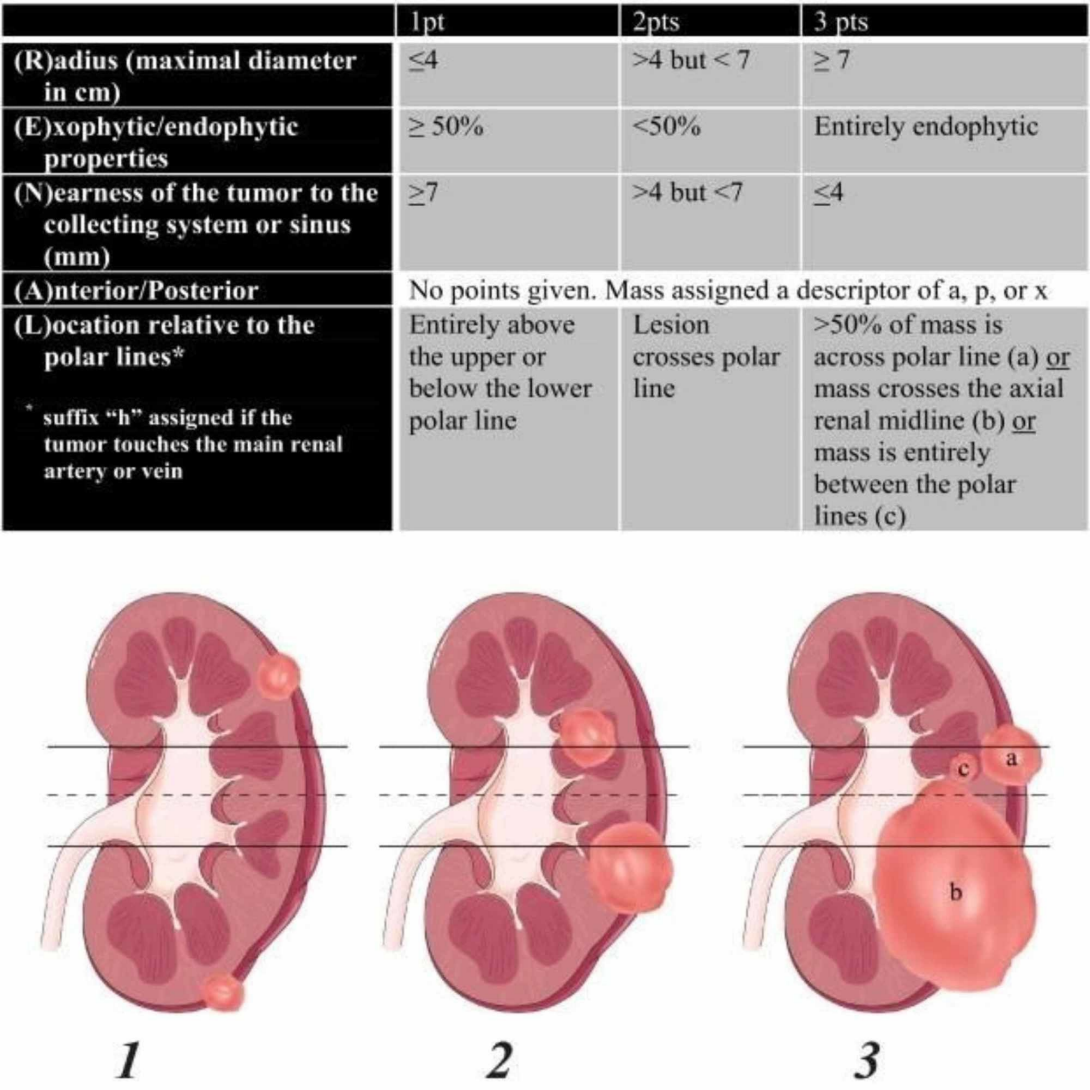
RENAL nephrometry score with the scoring of individual components Polar lines (solid lines) and axial renal midline (broken line) are shown on each sagittal view of the kidney. Numbers 1 to 3 represent points attributed to each category of the lesion. * pts = points; RENAL: radius, exophytic/endophytic properties, nearness of tumor to the collecting system or sinus in millimeters, anterior/posterior, location relative to polar lines Adapted from Kutikov A, Uzzo RG. The R.E.N.A.L. nephrometry score: a comprehensive standardized system for quantitating renal tumor size, location and depth. J Urol. 2009; 182:844-853

Radiologic features of renal masses were evaluated and scored using RENAL-NS. A three-point scale was used for each RENAL component except for “A,” to which we added the suffix “a” for the anterior location, “p” for the posterior location, and “x” when the location was indeterminate. In addition, the suffix “h” was used in order to designate a hilar location if the tumor abutted the main renal artery or vein. The polar lines were drawn as shown in Figure 1 (taken from the article by Kutikov et al.) [1]. After all the points had been summed, tumors were classified as low complexity (4-6 points), intermediate complexity (7-9 points), or high complexity (10-12 points) (Figure 1).

Imaging characteristics of all cases were evaluated by two body imaging radiologists with at least five years of experience at our center who independently evaluated and scored all lesions. Both radiologists were blinded to patient management and outcomes.

Demographic features, radiographic characteristics of the renal masses prior to surgery, surgical or interventional modalities, clinical and pathological stage, total RENAL-NS, and individual anatomic descriptor components were also summarized.

Statistical analysis was conducted using the Statistical Package for the Social Sciences (SPSS) 19.0 (IBM Corp., Armonk, NY). Cohen’s Kappa statistics were used to assess the interobserver agreement for the final total RENAL-NS system scores. Positive Kappa values can range from 0 to 1; the former indicating a lack of agreement and the latter indicating perfect agreement. Agreement was considered slight at values = 0.20, fair at values from 0.21 to 0.40 fair, moderate at values from 0.41 to 0.60, substantial at values from 0.61 to 0.80 high, and almost perfect at values ≥ 0.81. P < 0.05 was considered statistically.

## Results

The mean patient age was 59.9 years, ranging from 34 to 88 years. Of the 49 patients evaluated, 34 (69.4%) were male and 15 were female (30.6%). Lesions were located in the right kidney in 28 patients (57.1%) and in the left kidney in 21 patients (42.9%). The lesions were malignant renal cell carcinomas in all cases. The mean longest axis of the tumors was 7.9 cm, ranging from 1.7 to 24.5 cm. The mean volume of the lesion was 426 cm^3^, ranging from 4.1 to 2894 cm^3^ (Table 1).

**Table 1:** CT imaging features, pathology, and outcome of all lesions RENAL: radius, exophytic/endophytic properties, nearness of tumor to the collecting system or sinus in millimeters, anterior/posterior, location relative to polar lines; RCC: renal cell carcinoma; T: tumor; N: nodes; M: metastases

RENAL-Nephrometry Score class	
Low complexity	6 (12.2%)
Intermediate complexity	17 (34.7%)
High complexity	26 (53.1%)
Venous thrombosis	
No venous invasion	34 (69.4%)
Renal vein	4 (8.2%)
Infradiaphragmatic inferior vena cava	7 (14.3%)
Supradiaphragmatic inferior vena cava	4 (8.2%)
Pathological staging	
T1a	11 (22.4%)
T1b	11 (22.4%)
T2a	4 (8.2%)
T2b	4 (8.2%)
T3a	7 (14.3%)
T3b	7 (14.3%)
T3c	2 (4.1%)
T4	3 (6.1%)
N0	40 (81.6%)
N1	9 (18.4%)
M0	33 (67.3%)
M1	16 (32.7%)
Histopathology	
Clear cell RCC	40 (81.6%)
Papillary cell RCC	4 (8.2%)
Chromophobe RCC	2 (4.1%)
RCC with sarcomatoid features	3 (6.1%)
Histology grade (Fuhrman Grade)	
1	3 (6.1%)
2	31 (63.3%)
3	12 (24.5%)
4	3 (6.1%)
Interventional procedure	
Partial nephrectomy	10 (20.4%)
Radical nephrectomy	21 (42.9%)
Cytoreductive nephrectomy	16 (32.7%)
Radiofrequency ablation	1 (2%)
Angioembolization	1 (2%)

Interobserver agreement for each of the RENAL-NS parameters, respectively, and overall complexity was calculated. The total agreement was 82%, 51%, 84%, 69%, 73%, and 90%, corresponding to the Kappa values of 0.72, 0.33, 0.44, 0.49, 0.58, and 0.83, respectively. The radius, nearness to the collecting system, and overall complexity showed the best agreement. Overall complexity showed almost perfect agreement. The exophytic properties of the lesion showed the least agreement (Table 2).

**Table 2:** Interobserver reliability of RENAL nephrometry score between two radiologists and the Kappa values RENAL: radius, exophytic/endophytic properties, nearness of tumor to the collecting system or sinus in millimeters, anterior/posterior, location relative to polar lines

Statistic	Radius	Exophytic	Nearness	Anterior/Posterior	Location	Complexity
Kappa	0.72	0.33	0.44	0.49	0.58	0.83
p-value	<0.001	0.01	<0.001	<0.001	<0.001	<0.001
Agreement	40/49 (82%)	25/49 (51%)	41/49 (84%)	34/49 (69%)	36/49 (73%)	44/49 (90%)
95% CI	0.56-0.88	0.12-0.54	0.19-0.69	0.28-0.70	0.41-0.75	0.69-0.97

## Discussion

The biology of RCC is often heterogeneous. Even though approximately 30% of all renal tumors present with systemic disease, many localized renal masses appear to follow a relatively indolent clinical course [5]. Cross-sectional imaging is important for diagnosis and staging and for the assessment of response to therapy in patients with renal cell carcinoma [6].

**Figure 2:**
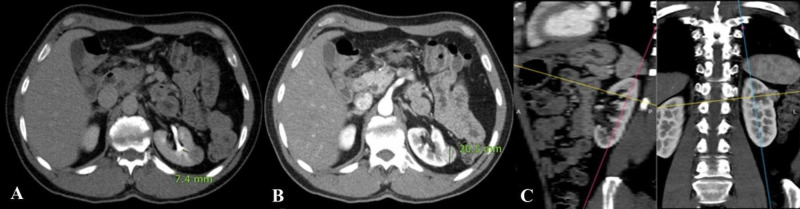
Low complexity lesion (5p) according to the RENAL nephrometry score A) Excretory phase of a triphasic CT scan showing left renal cell carcinoma, which is 7.4 mm away from the renal collecting system. B) Arterial phase contrast-enhanced CT scan showing the maximum diameter of the lesion measuring 20.5 mm. C) Sagittal and coronal reformatted sections showing the lesion location entirely in the upper pole. RENAL: radius, exophytic/endophytic properties, nearness of tumor to the collecting system or sinus in millimeters, anterior/posterior, location relative to polar lines

**Figure 3:**
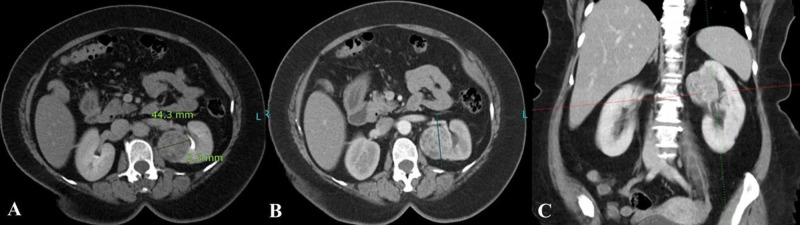
Moderate complexity lesion (8p) according to the RENAL nephrometry score A) Excretory phase of the triphasic CT scan showing a left renal lesion with a diameter of 4.4 cm, which is 2 mm away from the renal collecting system. (B) Venous phase contrast-enhanced CT scan showing > 50% of the lesion being exophytic. (C) Coronal reformatted image showing the lesion crossing the polar line. RENAL: radius, exophytic/endophytic properties, nearness of tumor to the collecting system or sinus in millimeters, anterior/posterior, location relative to polar lines

**Figure 4:**
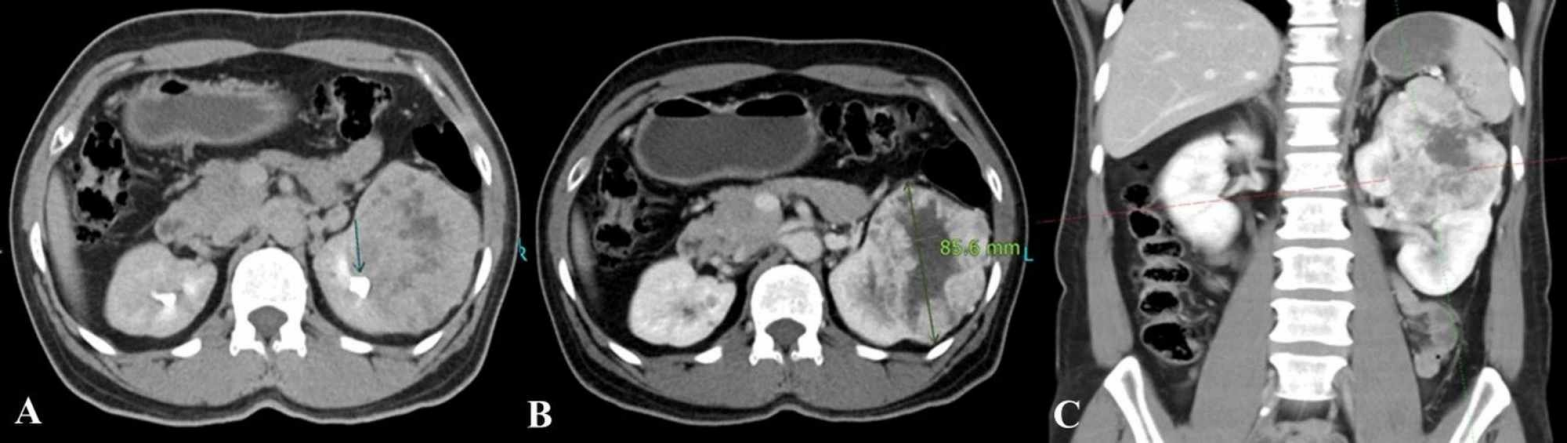
High complexity lesion (score 11x) according to the RENAL nephrometry score A) Excretory phase of the triphasic CT scan showing a left renal lesion abutting the renal collecting system. B) Venous phase axial CT showing the same lesion diameter of 8.5 cm. C) Venous phase coronal reformatted image shows <50 % of the lesion being exophytic and crossing the axial midline. RENAL: radius, exophytic/endophytic properties, nearness of tumor to the collecting system or sinus in millimeters, anterior/posterior, location relative to polar lines

Various models have been proposed to classify renal tumors, however, they have had limited success in reliably and consistently characterizing tumor anatomy. The RENAL-NS is based on the five most reproducible features that characterize the anatomy of a solid renal mass on contrast-enhanced cross-sectional imaging [1]. In addition to greater surgical complications (such as postoperative bleed, ischemia, and urologic complications), higher nephrometry scores have been shown to correlate with pathological stage, nuclear (Fuhrman) grade, and mortality from renal cell carcinoma [7]. In prior studies, the frequency of T3 lesions has been reported between 2.7% and 25%, with the frequency in our sample being 53.1%, indicating that our study population had lesions with higher complexity [8]. Lesions of low and moderate complexity were adequately represented in our study population, in six (12.2 %) and 17 (34.7 %) patients, respectively. Therefore, an inference can be confidently drawn that the RENAL-NS system produces scores that are reproducible and show good agreement, regardless of lesion complexity (Figures 2-4).

The reliability assessed showed the concordance among two observers to be 82%, 51%, 84%, 69%, 73%, and 90% for the R, E, N, A, and L components, The corresponding Kappa values for each of these five components were 0.72, 0.33, 0.44, 0.49, 0.58, and 0.83, respectively. Our findings demonstrate high to perfect agreement for the R, N, and L components of the scoring system between two radiologists; these findings were consistent with data from prior studies [7-10]. These results have been shown to be important, as tumor size (radius) is considered a key feature for planning surgical techniques [11-12], and nearness to the collecting system may predict complications of nephron-sparing surgery [13-14].

The exophytic properties of renal lesions showed the least agreement in our study. This is for the cases that were discordant in terms of the final score, however, no significant implications in the surgical plan or outcome were observed. This specific result was, however, contrary to findings from prior studies, which showed a 92%-98% agreement with a Kappa of 0.87-0.96 [7-10]. One possible explanation for this may be the usage of different reconstruction planes (like axial, coronal, and sagittal) viewed for interpretation by the two radiologists.

This study has a few limitations of note. Because of the retrospective nature, there are potential selection biases that may have been inherently involved. Due to the single-center experience, the results may not be fully generalizable to other populations and the sample size included was also not large enough, which caused wide 95% confidence intervals around some of our estimates. We only included imaging analysis done by two independent reviewers. Finally, we did not assess the efficacy of the RENAL-NS system for predicting the type of renal surgery or grade of the tumor and we also did not statistically determine whether ultimately surgical planning was carried out according to the RENAL-NS prediction.

However, we must emphasize that clinicians’ familiarity with the nephrometry scores is on the rise as evidenced by data obtained by clinical research. In one such study, patients with 81% of unresectable RCC were categorized as high complexity, and the post neoadjuvant of 46% was downgraded to moderately complex, which aided in surgery [15]. Another study by urologists of our institute also implemented the RENAL-NS to investigate the potential effect of off-clamp vs. hilar clamping partial nephrectomy on renal function [16]. Therefore, our data highlight the fact that the RENAL-NS system is an easy method to assess the complexity of renal tumors while facilitating treatment decision-making by multidisciplinary teams. The scoring system also provides a method of standardizing academic reporting by radiologists. We feel that overall complexity agreement is the most decisive factor for the outcome rather than the individual features of the scale.

## Conclusions

The results of our study showed substantial agreement for each of the individual components and the overall RENAL-NS system scores between two body imaging radiologists. The best results were found for tumor size (radius), nearness to collecting system, and complexity of the tumor. We, therefore, conclude that the RENAL-NS is a helpful tool to evaluate the anatomical features of renal tumors, and it is simple and easily reproducible for radiologists and urologists.
